# The rs2619566, rs10260404, and rs79609816 Polymorphisms Are Associated With Sporadic Amyotrophic Lateral Sclerosis in Individuals of Han Ancestry From Mainland China

**DOI:** 10.3389/fgene.2021.679204

**Published:** 2021-08-06

**Authors:** Jie Zhang, Weiwen Qiu, Fan Hu, Xiong Zhang, Youqing Deng, Hongbing Nie, Renshi Xu

**Affiliations:** ^1^Department of Neurology, The First Affiliated Hospital of Nanchang University, Nanchang, China; ^2^Department of Neurology, The Affiliated People’s Hospital of Nanchang University, The First Affiliated Hospital of Nanchang Medical College, Jiangxi Provincial People’s Hospital, Nanchang, China; ^3^Department of Neurology, Maoming People’s Hospital, Maoming, China; ^4^Department of Neurology, The Third Affiliated Hospital of Nanchang University, Nanchang, China

**Keywords:** genetics, single nucleotide polymorphism, pathogenesis, amyotrophic lateral sclerosis, Chinese Han ancestry population

## Abstract

The pathogenesis of sporadic amyotrophic lateral sclerosis (sALS) remains unknown; however, recent research suggests that genetic factors may play an important role. This study aimed at investigating possible genetic risk factors for the pathogenesis of sALS. In our previous study, we conducted a genome-wide association study (GWAS) in 250 sALS patients and 250 control participants of Han ancestry from mainland China (HACM) and retrospectively analyzed the previously reported candidate loci related with sALS including our GWAS investigated results. In this study, twenty-seven candidate loci that were most likely associated with sALS were selected for further analysis in an independent case/control population of 239 sALS patients and 261 control subjects of HACM ethnicity using sequenom massARRAY methodology and DNA sequencing. We discovered that the polymorphism rs2619566 located within the contactin-4 (*CNTN4*) gene, rs10260404 in the dipeptidyl-peptidase 6 (*DPP6*) gene, and rs79609816 in the inositol polyphosphate-5-phosphatase B (*INPP5B*) gene were strongly associated with sALS in subjects of HACM ethnicity. Subjects harboring the minor C allele of rs2619566 and the minor T allele of rs79609816 exhibited an increased risk for sALS development, while carriers of the minor C allele of rs10260404 showed a decreased risk of sALS development compared to the subjects of other genotypes. The polymorphisms of rs2619566, rs10260404, and rs79609816 may change or affect the splicing, transcription, and translation of *CNTN4*, *DPP6*, and *INPP5B* genes and may play roles in the pathogenesis of sALS roles in the pathogenesis of sALS.

## Introduction

Amyotrophic lateral sclerosis (ALS) is the most common adult-onset motor neuron disease. It is characterized by the progressive neuronal loss and degeneration of upper motor neurons and lower motor neurons. The death of motor neurons causes a loss of the ability of central nervous system (CNS) to control voluntary muscle movements, contributing to the development of progressive atrophy of voluntary muscles, eventually resulting in death of the patient due to respiratory failure in the later stages of the disease. Most ALS patients succumb to the disease within 3–5 years after disease onset. Although the patient may or may not present with a family history of the disease, ALS is divided into familial ALS (fALS) and sporadic ALS (sALS) (1). Several genes and/or loci associated with risk of fALS development have been identified in the recent years. Although certain genetic loci have been associated with sALS risk, the exact genetic mechanism for sALS has not been elucidated, and sALS is hypothesized to possess a more complex pathogenesis. The reasons for progressive and selective motor neuron death occurring in sALS remain elusive. The pathogenesis of sALS remains an enigma (Ludolph et al., 2012).

The recently acknowledged pathogenesis of sALS is mainly focused on environmental and genetic factors. Potential environmental factors predisposing to sALS development include viral and bacterial infections (Sher, 2017; Yu and Pamphlett, 2017; Xue et al., 2018), organophosphate, organochlorine (Su et al., 2016; Riancho et al., 2018; Lian et al., 2019), and heavy metal exposure (Callaghan et al., 2011; Garzillo et al., 2015; Peters et al., 2017; Riancho et al., 2018), intense physical activity (Tsitkanou et al., 2019), smoking, electromagnetic fields, electric shocks, cyanotoxins, and military service (Vinceti et al., 2017; Swash and Eisen, 2020). However, none of the known environmental risk factors has been conclusively determined, and no firm conclusions have been deduced thus far (Longinetti and Fang, 2019). If environmental factors are truly a causal risk factor in sALS development, the genetic susceptibility would be expected to increase the risk of sALS development due to exposure to environmental agents (Nowicka et al., 2019). Therefore, genetic factors have garnered considerable attention in the study of sALS pathogenesis since the discovery of *SOD1* mutations in sALS. During the last decade, the evolution of molecular genetic technologies has rapidly advanced our knowledge concerning the genetic pathogenesis of sALS. The development of fALS has been attributed to mutations in at least 24 different genes. Certain mutations responsible for fALS development have been identified also in patients with sALS (Chia et al., 2018; Mathis et al., 2019). Recent large-scale parallel sequencing technologies have facilitated disease-gene discovery, rare variants in more than 50 genes have now been identified to be associated with sALS. Thus, sALS has been considered a complex gene-related disease (Van Doormaal et al., 2017).

Genetic testing may aid the exploration of mutations in ALS-related genes. However, the loci that are most likely to be affected in individual patients with sALS cannot be easily predicted (Yousefian-Jazi et al., 2020). Although a series of possible ALS-related genes and mutant loci have been successively reported in the literature, currently there is no single gene or mutant locus that can completely explain the pathogenesis of ALS (Morgan and Orrell, 2016; Yousefian-Jazi et al., 2020). Therefore, it has been suggested that the sALS pathogenesis is associated with multiple genes and/or mutant loci. To this end, the investigation of additional ALS-related genes and/or mutant loci is extremely important.

In this study, we selected the 27 loci that were most likely to be associated with sALS development based on the results of the present study including our GWAS study of 250 sALS and 250 controls as well as other related previous studies (Van Es et al., 2007, 2008; Laaksovirta et al., 2010; Fogh et al., 2014; Van Doormaal et al., 2014; Xie et al., 2014), and we further analyzed them in an independent cohort of the 239 individuals with sALS and the 261 controls of Han ancestry from mainland China (HACM). We discovered that the polymorphism of the rs2619566 in contactin 4 (*CNTN4*), the rs10260404 in dipeptidyl-peptidase 6 (*DPP6*), and the rs79609816 in inositol polyphosphate-5-phosphatases B (*INPP5B*) were markedly associated with sALS development in the subjects of HACM ethnicity. These results provide evidence to a certain extent for the further elucidation of the pathogenesis of sALS.

## Materials and Methods

### Subjects

The sALS dataset was developed based on the data obtained by combining the participants from two affiliated university hospitals, namely The First Affiliated Hospital of Nanchang University and the Affiliated Guangdong General Hospital of Nan fang Medical University. All sALS and control subjects were recruited from HACM in the southern regions of China (Jiangxi and Guangdong Province). A signed informed consent was obtained from all participants in the study. sALS diagnosis was performed according to the El Escorial criteria of the World Federation of Neurology (Brooks et al., 2000). All subjects were subjected to the same evaluation, which included a medical history, a Mini Mental State Exam, a review of the family history of ALS, related disorders in first-degree relatives, toxicant exposure associated with sALS development, biochemical tests, and brain and spinal magnetic resonance imaging to exclude the presence of other neurological diseases that might mimic the clinical presentation of ALS (e.g., tumors, demyelination disorders, hydrocephalus, cervical myelopathy, and others).

The studied populations were composed of a total of 489 sALS cases and 511 controls, of which 250 sALS cases and 250 controls were included in the genome-wide association study (GWAS) analysis (Xie et al., 2014), and 239 sALS cases and 261 controls were included in the polymorphism analysis of the 27 most likely candidate loci for sALS development. The male/female ratio was 143/96 for the sALS cases, and was 148/113 for the controls. The mean (range) age was 47 (45–65) years for the sALS cases, and was 65.7 (65–75) years for the controls. Gender was a significant variable because sALS seemed to affect more men than women, and our data exhibited a significant gender disparity. The control subjects were older than the patients, and this aspect was included in the study design to minimize the chance that the control subjects were too young to have developed the disease. Nevertheless, we controlled for age at enrollment to avoid confounding by age-related factors, and the early- and late-onset sALS patients were excluded. The entire clinical disease course spanned across 3–5 years, which precluded the development of rapid and slow progressive sALS, and the cases with atypical sALS clinical manifestations were removed. Thus, sALS patients with typical age, clinical course, and phenotype were enrolled in this study. Based on the current studied information, the genetic pathogenesis about sALS should be involved in some complex and multiple genes and mutations, isn’t sole gene or mutation to contribute to sALS. Therefore, we didn’t perform the genetic testing of the known ALS-related genes to exclude whether or not the presently known genes and mutations existed in our sALS patients and control subjects.

### Selection of Single Nucleotide Polymorphisms (SNPs)

In our previous pooling GWAS performed by inclusion of 250 sALS patients and 250 control subjects from HACM, we revealed that the 7 loci, namely rs79609816 and rs62172104 in *INPP5B*, rs9825420 in *ITGA9*, rs2685056 in *ALCAM*, rs7117082 in *OPCML*, rs9329300 in *PFKP*, and rs11061269 in *GPR133*, were strongly associated with sALS development in subjects of the HACM origin (the significance threshold was *p* < 5.8 × 10^−8^), and these loci were not reported previously (Xie et al., 2014). Moreover, we retrospectively analyzed the previously reported candidate loci related with pathogenesis of sALS, and found that 20 SNPs, namely rs62484656 in *CSMD1*, rs17722673 in *HECW1*, rs882467 in *DPP6*, rs3812208 in *ATXN1*, rs28461450 in *LIPC*, rs79591932 in *RBMS1*, rs9907321 in *SLC39A11*, rs4964009 in *ITPR2*, rs34517760 in *SOD1*, rs13065219 in *CNTN4*, rs10260404 in *DPP6*, rs697739 in *ATXN1*, rs3825776 in *LIPC*, rs10192369 in *RBMS1*, rs8066857 in *SLC39A11*, rs2306677 in *ITPR2*, rs13048019 in *SOD1*, rs2619566 in *CNTN4*, rs16856202 in *DISC1*, and rs34517613 in *KRT18P55*, were susceptibility loci for sALS, which were strongly associated with sALS development in the previous studies (Van Es et al., 2007, 2008; Laaksovirta et al., 2010; Fogh et al., 2014; Van Doormaal et al., 2014; Xie et al., 2014). Therefore, in this study, 27 SNPs were selected to further ascertain their association with the development of sALS (Table 1).

**TABLE 1:** All SNPs were chosen in the association study.

Our previous pooling GWAS scanning						Previous researches			
Gene	Chro	SNP ID	Position	OR (95%CI)	*P*-value	SNP ID	Position	OR (95%CI)	*P*-value
*INPP5B*	1	kgp15327256 (rs79609816)	38348765	0.057347 (0.115019–0.028593)	2.24 × 10^–8^				
*ITGA9*	3	rs9825420	37604012	3.033502 (4.049382–2.272478)	2.55 × 10^–8^				
*ALCAM*	3	rs2685056	104418573	2.581274 (3.434586–1.939965)	4.00 × 10^–8^				
*OPCML*	11	rs7117082	133392294	0.358089 (0.489104–0.262168)	8.43 × 10^–9^				
*PFKP*	10	rs9329300	2789594	0.322536 (0.444649–0.233959)	2.46 × 10^–9^				
*GPR133*	12	rs11061269	131456449	0.264827 (0.402259–0.17435)	8.45 × 10^–10^				
*INPP5B*	2	kgp8851185 (rs62172104)	77015974	MD	2.06 × 10^–8^				
*CSMD1*	8	kgp12078483 (rs62484656)	4754792	MD	2.42 × 10^–7^				
*HECW1*	7	kgp12304308 (rs17722673)	43178332	MD	3.50 × 10^–3^				
*DPP6*	7	rs882467	154701338	MD	5.25 × 10^–4^	rs10260404^a^	154210798	1.3 (1.18–1.43)	5.00 × 10^–8^
*ATXN1*	6	kgp8327591 (rs3812208)	16704445	MD	7.00 × 10^–4^	rs697739^2b^	16742033	2.04 (1.18–2.90)	4.00 × 10^–6^
*LIPC*	15	kgp8216028 (rs28461450)	58693661	MD	7.45 × 10^–4^	rs3825776^a^	58746830	1.34 (1.20–1.46)	9.00 × 10^–6^
*RBMS1*	2	kgp14738211 (rs79591932)	161428820	MD	4.94 × 10^–5^	rs10192369^b^	161380888	1.17 MD	9.00 × 10^–6^
*SLC39A11*	17	kgp13969888 (rs9907321)	70977240	MD	2.12 × 10^–5^	rs8066857^b^	70696103	1.48 MD	8.00 × 10^–6^
*ITPR2*	12	kgp3041552 (rs4964009)	26798095	MD	1.75 × 10^–4^	rs2306677^c^	26636386	1.58 (1.30–1.91)	3.00 × 10^–6^
*SOD1*	21	kgp10760302 (rs34517760)	32985381	MD	1.02 × 10^–4^	rs13048019^d^	32918294	2.02 (1.61–2.53)	3.00 × 10^–8^
*CNTN4*	3	kgp11325216 (rs13065219)	2146481	MD	7.50 × 10^–4^	rs2619566^b^	2624938	3.03 (1.71–4.35)	7.00 × 10^–6^
*DISC1*	1					rs16856202^b^	232155151	2 MD	8.00 × 10^–6^
*KRT18P55*	17					rs34517613^e^	26610252	0.822 (0.769–0.879)	1.11 × 10^–8^

*Chro, Chromosome; SNP, Single nucleotide polymorphism; GWAS, Genome wide association study; OR, Odds ratio; CI, Confidence interval; MD, Missing Data. ^*a*^Van Es et al. (2008), ^*b*^Landers et al. (2009), ^*c*^Van Es et al. (2007), ^*d*^Laaksovirta et al. (2010), ^*e*^Fogh et al. (2014).*

### SNP Genotyping by Using the Sequenom MassARRAY Technology

Genotyping was performed using the sequenom massARRAY platform (Sequenom, San Diego, California, United States) according to the manufacturer’s instructions. The selected 27 SNPs were genotyped as part of a sequenom plex, which enabled high-throughput multiplexing of the assays into a single well. For quality control, 5% of the samples were subjected to the repeated genotyping, and the results showed 100% consistency.

### Main Apparatus and Reagent

Amplification instrument: ABI GeneAmp^®^ 9700 384 dual, mechanical arm, massARRAY nanodispenser RS1000. Analyzer: massARRAY compact system. Reagents: The Complete genotyping reagent kit for massARRAY^®^ compact 384.

### Polymerase Chain Reaction (PCR) Using 384-Well Plates

A PCR cocktail solution was prepared by combining 1.8 μL ddH_2_O, 0.5 μL 10 × PCR Buffer, 0.1 μL dNTPs, 0.2 μL PCR enzyme (5 U/μL), 1 μL primer mix (0.5 μM), 0.4 μL 25 mM MgCl2, and 1 μL DNA template, to obtain a total volume of 5 μL. One-μL volume of the appropriate genomic DNA (5–10 ng/μL) was added into each well of a 384-well microtiter plate (Marsh Biomedical Products, Inc. #SP 0401 Sequenom). Four-μL volume of the PCR cocktail solution was dispensed into each well of the 384-well microtiter plate, followed by centrifugation of the microtiter plate at 1,000 RPM for 1 min. Subsequently, contents in the microtiter plate were gently mixed and were re-centrifuged before conduction of PCR. PCR was conducted using the 384-well microtiter plate as per the following amplification conditions: 94°C denaturation for 20 s, 56°C annealing for 30 s, 72°C extension for 1 min, for a total of 45 cycles. The primers described in Table 2 were used for conducting the PCR reactions for this study. This general PCR protocol using a 384-well microtiter plate was applied to the different PCR amplifications performed in this study.

**TABLE 2:** Primer sequences genotyped by sequenom.

SNP ID	Forward primer	Reverse primer	Unextended primer	Unextended Direction
rs28461450	ACGTTGGATGGCAAATCTAGTAGCCAGGTG	ACGTTGGATGCAAGAACAGAACCAACTGGG	CCACCCCATCAAAGT	Forward
rs8066857	ACGTTGGATGGGTAGAGACCCACACCAAAA	ACGTTGGATGAGGTACCAGAAGTACCACTC	GCCAGCTCCTGTGTT	Reverse
rs11061269	ACGTTGGATGGGTGCCATGCCATCAATTTC	ACGTTGGATGAACAGACACAGCGATGTCAC	GGACAGGGCCGTGGC	Forward.
rs3812208	ACGTTGGATGGAGGTGCGCACTGAGTTAAA	ACGTTGGATGCTGTACTTAGCAGGCTCTGG	GCTCTGGAGACCAAAT	Reverse
rs882467	ACGTTGGATGTGCTGTGGAAGGCTGTCTG	ACGTTGGATGAGATCTGCCACACGAAGTAG	ACGAAGTAGGACGGAT	Reverse
rs34517613	ACGTTGGATGCCTGAGGTTAAAATTGAGTGG	ACGTTGGATGCTTTCTTTTTCCCTGGACAC	GACACCAATTTCCATCC	Forward.
rs3825776	ACGTTGGATGTTTTACTCCCACATGGTGAC	ACGTTGGATGAAGCCTCTTGTGTGTAGCAG	ACCCCACCTGTTGACTAGA	Forward.
rs2685056	ACGTTGGATGAGCTAAGTGTCAGGTAAAGG	ACGTTGGATGGTAGCAGAAGCCAGGATTAC	AGCCAGGATTACTGGACTC	Forward.
rs79591932	ACGTTGGATGATGTTCTTTGGCCACGTTCC	ACGTTGGATGCCTGGATTAGTTAGGGCTTG	gggaTTAGGGCTTGGCTCTG	Reverse
rs4964009	ACGTTGGATGCAGTTGTGTTCCTCAGGAAG	ACGTTGGATGGTTGATCCTAAGAGCTCTCC	tcCTTCTTCATGCTAATCCTC	Forward.
rs16856202	ACGTTGGATGTACAGGTTGCTCGGCATTTC	ACGTTGGATGTGAATTGCTGAAAGGACCGC	tGACCGCCTGACAAGACAAAT	Forward.
rs17722673	ACGTTGGATGTGGAAAGCGCTTTGGAATAC	ACGTTGGATGCCCTCTCTCTCTTTTAGACC	tcacACCTCAAGCAACAGTTTT	Forward.
rs62484656	ACGTTGGATGTTTCCGTTTTTTTCTTACC	ACGTTGGATGCGTCTGTTTTGTTTCTACTG	TTTCATCGTATCAACCAAAAAT	Forward.
rs7117082	ACGTTGGATGGTAATGATCTGGGAGACTTC	ACGTTGGATGGACACGTATCTGGCATTTGG	agggTCTTCTGCATATCAAGGA	Forward.
rs9825420	ACGTTGGATGCCCCGAAAGATCAGGGAAAA	ACGTTGGATGGCATCTTCCCTACCACTGTC	ccccCTGTCGCTGATGAGGAGCA	Reverse
rs34517760	ACGTTGGATGGGAAGAAAGCATAAGGAGGG	ACGTTGGATGTTGAGCTTCTGTGAAGCGTG	agcggGAATCACTGAGTGTGAGT	Forward.
rs2306677	ACGTTGGATGGTCCCATGAAAATGTTCAAG	ACGTTGGATGATGAAAACTGGGTGGTGGTC	gtgaTGGGTGGTGGTCATGACAC	Forward.
rs9329300	ACGTTGGATGGAGAATCCTTTCCTCCAACG	ACGTTGGATGGGTGACCTCATCTGTTTCTG	gGTTCCTATTTCTACTGGAGTGTT	Forward.
rs13065219	ACGTTGGATGTGCTCTCTTTTCCCCATACC	ACGTTGGATGAACTAGCCAGTCTTTGTCAC	cccatCCAGTCTTTGTCACAGGTTT	Forward.
rs2619566	ACGTTGGATGTGAAGATCTGGCCATGGTTG	ACGTTGGATGCTGTGAAATGCTCCCTGTTG	aatgaTCCCTGTTGAAGTAACATAT	Forward.
rs697739	ACGTTGGATGACTCACATTCTGCTAATCAC	ACGTTGGATGCTTTAATTTTCCTCCCAGCG	gttatCGAGCTGCAAAGCTGTTTCA	Reverse
rs79609816	ACGTTGGATGTTTTTTTCTCTCGCCATCCC	ACGTTGGATGCTAAGGAGAACACAAGGCTG	gtgggCCAAGATAATTGAGCAAGCA	Forward.
rs10260404	ACGTTGGATGACTGATTCCACCACAAGCTC	ACGTTGGATGGAAGGAAACTGTCCTCATAC	tcctCTATCTTGCTGTGTTGACATAC	Reverse
rs9907321	ACGTTGGATGTGGTAGGCACAATCACCAAG	ACGTTGGATGACTATTTTGGATATATTTGG	ATGTTATTACAATTAATTTCACCTGTT	Reverse
rs62172104	ACGTTGGATGAGGACTCAAACATAGAGAAG	ACGTTGGATGACTATAAACCCCATAGACAC	cccacACCCCATAGACACAAAAACATTT	Reverse
rs10192369	ACGTTGGATGCATAATCCCGACCCTCATAG	ACGTTGGATGACCACCATCTGACCTACTAC	aACATTTTATGTATTATGTCTTTCTTCT	Reverse
rs13048019	ACGTTGGATGTTCTCTAACTTTGAAAGATT	ACGTTGGATGCCCATTCAAAGATGAAGGTG	ggggCTGATTATACAAGTAACTACTACT	Forward.

### Preparation of Shrimp Alkaline Phosphatase (SAP) Enzyme Solution and Conduction of the SAP Reaction of PCR Products

The SAP enzyme solution was prepared in a 1.5-mL tube as by combining 1.53 μL RNase-free ddH_2_O, 0.3 μL SAP enzyme, and 0.17 μL SAP buffer, in a total volume of 2 μL. The 1.5-mL tube containing the SAP enzyme solution was subjected to vortexing for 5 s, and was then subjected to centrifugation for 10 s at 5,000 RPM. Subsequently, 2 μL of the SAP enzyme solution was added to each well in the 384-well sample microtiter plate containing the PCR products, and the plate was sealed using a plate-sealing film. The 384-well microtiter plate was then subjected to centrifugation at 1,000 RPM for 1 min and incubated at 37°C for 40 min and at 85°C for 5 min.

### Preparation of High Plex iPLEX Gold Reaction Cocktail (Same Multiplexed Assays Performed for Different DNA Samples) and Conduction of the High Plex iPLEX Gold Reaction

The high plex iPLEX gold reaction cocktail solution was prepared in a 1.5-mL tube by combining 0.619 μL RNase-free ddH_2_O, 0.2 μL iPLEX Buffer Plus, 0.2 μL iPLEX termination mix, 0.94 μL iPLEX extend primer mix, and 0.041 μL iPLEX enzyme. A 384-well sample microtiter plate was centrifuged at 1,000 RPM for 1 min, after which 2 μL of high Plex iLEX gold reaction solution was added to each well, followed by the addition of 7 μL of PCR/SAP reaction solution, for a total of 9 μL volume per well. The 384-well sample microtiter plate with plate was sealed with sealing film, and centrifuged at 1,000 RPM for 1 min. The 384-well microtiter plate containing the samples was then subjected to a thermocycling reaction according to the following conditions: 94°C for 30 s, 94°C for 5 s, 52°C for 5 s, 80°C for 5 s, for 5 cycles, a total of 45 cycles, followed by incubation at 72°C for 3 min.

### Cleanup of the High Plex iPLEX Gold Reaction Products

The cleanup of high plex iPLEX gold reaction products involved the spreading of clean resin onto the 384-well plate containing the iPLEX products, addition of nanopure water to each well, rotation of the plate, and centrifugation of the 384-well plate.

### Spectra Acquisition for Genotyping Analysis

The ACQUIRE module controlled the massARRAY analyzer to acquire spectra from each SpectroCHIP.

### DNA Sequencing

For each SNPs, Sanger sequencing of a subset of samples was performed using ABI3500 (ABI3730xl; Applied Biosystems, Inc. CA) to confirm the genotyping results of sequenom massARRAY. Their nucleotide variants were analyzed using the DNASTARLaser gene software (Version v7.1) and compared with the DNA sequence information obtained from NCBI GenBank.

### Functional Prediction

The functional predictions for the intronic C/T polymorphism in the *CNTN4* gene (SNP rs2619566), the intronic C/T polymorphism in the *DPP6* gene (SNP rs10260404), and the intronic T/A polymorphism in the *INPP5B* gene (SNP rs79609816) were further analyzed. The prediction of binding sites and transcription factor binding sites was performed using the NHRscan (Sandelin and Wasserman, 2005) and Mscan (Alkema et al., 2004), respectively, the prediction of secondary structures was performed using Mfold (Zuker, 2003), and the prediction of binding sites for miRNA and long non-coding RNA (lncRNA) fragments was performed using Ensemble^1^ (version GRCh38). The 100-bp sequence both upstream and downstream of each SNP position was analyzed for the presence of the predicted binding sites for transcription factors using NHRscan. The 100-bp sequence both upstream and downstream of each SNP was also analyzed for prediction of the secondary structure. The 50-bp sequence upstream and downstream of each SNP was analyzed by sequence formatting, and was then aligned by using BLAST with the Ensemble non-coding RNA sequence to explore miRNA- and lncRNA-binding sites.

### Statistical Analysis

Statistical analysis was performed using the SPSS (Version 19.0) statistical software (SPSS, Chicago, IL, United States). The Hardy-Weinberg equilibrium (HWE) was first evaluated in the healthy controls. Pearson chi-square tests were used to compare the frequencies of alleles and genotypes in cases and controls. Minor allele frequency (MAF) and odds ratios (OR) with 95% confidence intervals (95% CI) were estimated to determine the role of each SNP in sALS risk. Two-tailed *p* < 0.05 were considered as statistically significant.

## Results

### Screening for Polymorphic Loci Associated With sALS by GWAS

In this study, we first performed a pooling GWAS for 250 sALS and 250 controls selected from the HACM population to screen for possible variant loci associated with the pathogenesis of sALS. The results showed that 7 novel SNPs (the rs79609816 and rs62172104 in *INPP5B*, the rs9825420 in *ITGA9*, the rs2685056 in *ALCAM*, the rs7117082 in *OPCML*, the rs9329300 in *PFKP*, and the rs11061269 in *GPR133*) were strongly associated with sALS in the HACM population (Xie et al., 2014). Secondly, we explored previously reported candidate loci associated with the pathogenesis of sALS, and revealed that 20 SNPs (the rs62484656 in *CSMD1*, the rs17722673 in *HECW1*, the rs882467 in *DPP6*, the rs3812208 in *ATXN1*, the rs28461450 in *LIPC*, the rs79591932 in *RBMS1*, the rs9907321 in *SLC39A11*, the rs4964009 in *ITPR2*, the rs34517760 in *SOD1*, the rs13065219 in *CNTN4*, the rs10260404 in *DPP6*, the rs697739 in *ATXN1*, the rs3825776 in *LIPC*, the rs10192369 in *RBMS1*, the rs8066857 in *SLC39A11*, the rs2306677 in *ITPR2*, the rs13048019 in *SOD1*, the rs2619566 in *CNTN4*, the rs16856202 in *DISC1*, and the rs34517613 in *KRT18P55*) were potentially associated with sALS (Table 1; Van Es et al., 2007, 2008; Laaksovirta et al., 2010; Fogh et al., 2014; Van Doormaal et al., 2014).

### Identification of the Genetic Association Between 27 SNPs and sALS Using the Sequenom MassARRAY Technology

**FIGURE 1:**
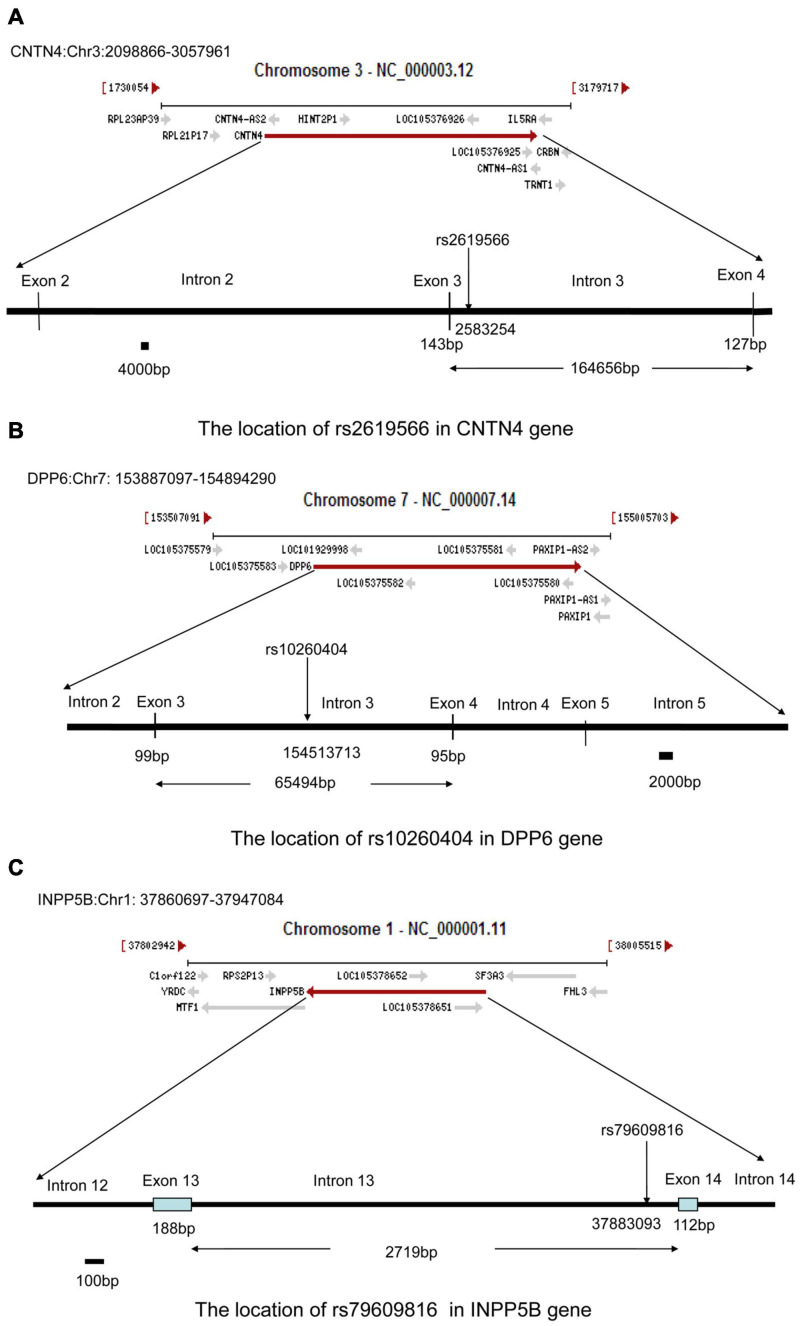
Locations of rs2619566 in the *CNTN4* gene, rs10260404 in the *DPP6* gene, and rs79609816 in the *INPP5B* gene. **(A)** The rs2619566 is located upstream of intron 3, adjoined to exon 3, at position 2583254 of the *CNTN4* gene on chromosome 3. **(B)** The rs10260404 is located in the middle of intron 3, at position 154513713 of the *DPP6* gene on chromosome 7. **(C)** The rs79609816 is located downstream of intron 13, adjoined to exon 14, at position 37883093 of the *INPP5B* gene on chromosome 1.

To further assess the association with sALS for the 27 candidate loci identified by GWAS, we performed sequenom massARRAY and DNA sequencing analyses using an independent cohort of 239 sALS cases and the 261 controls of Chinese ethnicity (Table 1). The following three novel SNPs were identified: the rs2619566 in the *CNTN4* gene (Chr 3:2583254) (Figure 1A), rs10260404 in the *DPP6* gene (Chr 7:154513713) (Figure 1B), and rs79609816 in *INPP5B* gene (Chr 1:37883093) (Figure 1C). The information of the 3 SNPs has been summarized in Tables 3, 4. All 3 SNPs were intronic polymorphisms. The minor allele frequencies (MAFs) of rs2619566 in the *CNTN4* gene and rs79609816 in the *INPP5B* gene were higher in the sALS patients (44.9 and 8.6%, respectively) than those in the controls (35.6 and 3.1%, respectively). The minor allele of rs2619566 (OR = 1.476, 95% CI = 1.143–1.906, *p* = 0.003) and rs79609816 (OR = 2.981, 95%CI = 1.649–5.387, *p* = 0.0003) significantly increased the risk of sALS development in HACM, suggesting these two polymorphisms might represent genetic susceptibility factors. Subjects harboring the minor C allele (CC + CT) of rs2619566 (*p* = 0.0003) and the minor T allele (TT + TA) of rs79609816 (*p* = 0.0016) exhibited an increased risk of developing sALS in comparison with the other genotypes (Table 3). The MAFs of rs10260404 in the *DPP6* gene were lower in the sALS patients (13.2%) than those in the controls (19.4%). The minor allele of rs10260404 (OR = 0.635, 95% CI = 0.450–0.895, *p* = 0.009) significantly decreased the risk of developing sALS in HACM, which represented a protective genetic factor. The carriers with the minor C allele (CC + CT) (*p* = 0.0284) had a significantly decreased risk of developing sALS in the HACM population (Table 3).

**TABLE 3:** Three SNPs shown the nominal significance at *P* < 0.05 in this study.

SNP	Group	Genotypes			Genotype		MAF	β	Allelic *P*-value	Allelic OR (95%CI)	Hap-Map HCB MAF
					χ^2^	*P*-value					
*CNTN4*		**CC**	**TT**	**CT**			**C Allele**				
rs2619566	Cases (*n* = 237)	37	61	139	16.41	0.0003	0.449	0.389	0.003	C Allele: 1.476 (1.143–1.906)	*C* = 0.395
	Controls (*n* = 257)	36	110	111			0.356	−0.389		T Allele: 0.677 (0.525–0.875)	
*DPP6*		**CC**	**TT**	**CT**			**C Allele**				
rs10260404	Cases (*n* = 238)	3	178	57	7.12	0.0284	0.132	−0.455	0.009	C Allele: 0.635 (0.450–0.895)	*C* = 0.209
	Controls (*n* = 258)	10	168	80			0.194	0.455		T Allele: 1.576 (1.118–2.222)	
*INPP5B*		**TT**	**AA**	**TA**			**T Allele**				
rs79609816	Cases (*n* = 238)	5	202	31	12.89	0.0016	0.086	1.092	0.0003	T Allele: 2.981 (1.649–5.387)	*T* = 0.025
	Controls (*n* = 261)	0	245	16			0.031	−1.092		A Allele: 0.335 (0.186–0.606)	

*SNP, Single nucleotide polymorphism; MAF, Minor allele frequency; OR, Odds ratio; CI, Confidence interval; HCB, Han Chinese in Beijing. P-value significance < 0.05.*

**TABLE 4:** Information of three SNPs associated with sALS.

Gene and description	SNP ID	Position	Function and SNP Type	Minor/Major allele
*CNTN4*, contactin 4	rs2619566	Chr 3: 2583254	Intron, Transition Substitution	C/T
*DPP6*, dipeptidyl-peptidase 6	rs10260404	Chr 7: 154513713	Intron, Transition Substitution	C/T
*INPP5B*, inositol polyphosphate-5-phosphatase B	rs79609816	Chr 1: 37883093	Intron, Transversion Substitution	T/A

### Confirmation of Results (SNP Positions in the CNTN4, DPP6, and INPP5B Genes) Based on the Sequenom MassARRAY Technology Using DNA Sanger Sequencing

**FIGURE 2:**
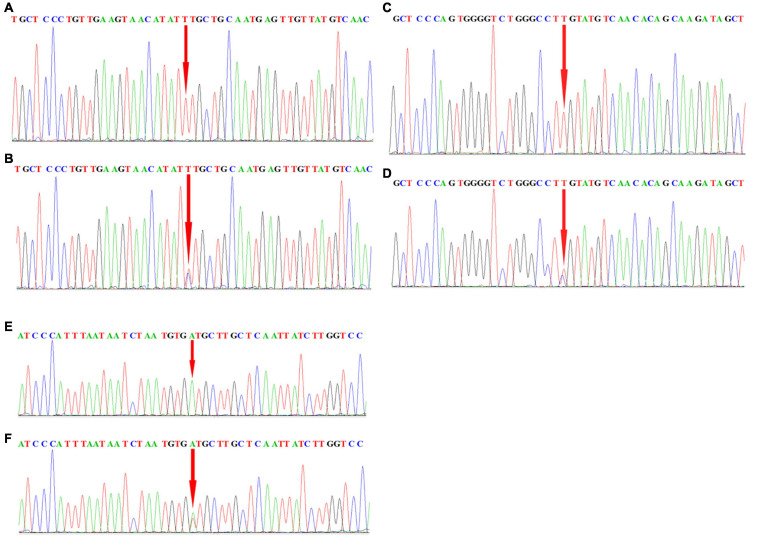
Sequencing data showing rs2619566 in *CNTN4*, rs10260404 in *DPP6*, and rs79609816 in *INPP5B*. **(A,B)** Peak graph showing the rs2619566 T > C variation in the *CNTN4* gene. **(C,D)** Peak graph showing the rs10260404 T > C variation in the *DPP6* gene. **(E,F)** Peak graph showing the rs79609816 A > T variation in the *INPP5B* gene. The variant loci are indicated by using a red arrow.

**FIGURE 3:**
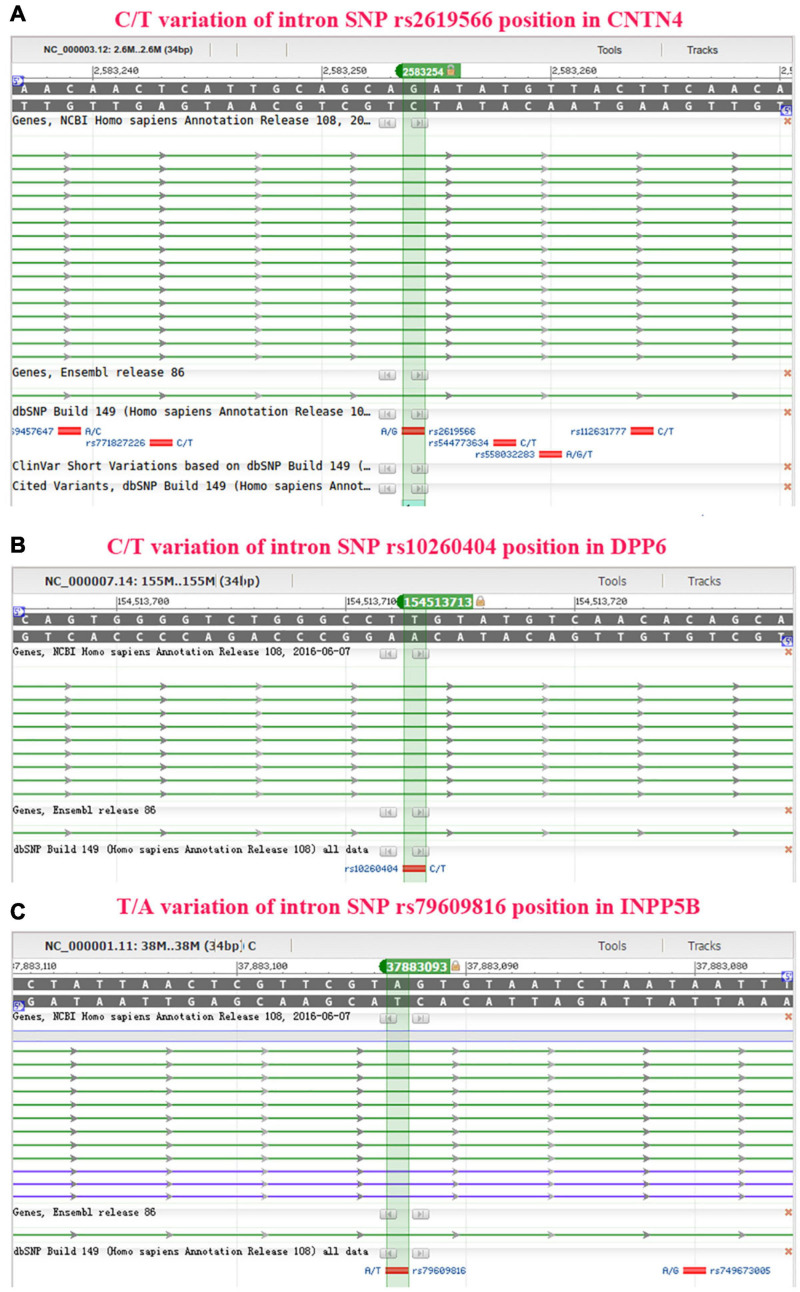
The rs2619566 variants in the *CNTN4* gene, rs10260404 variants in the *DPP6* gene, and rs79609816 variants in the *INPP5B* gene. **(A)** The rs2619566 T > C SNP site is shown in the *CNTN4* gene. **(B)** The rs10260404 T > C SNP site is shown in the *DPP6* gene. **(C)** The rs79609816 A > T SNP site is shown in the *INPP5B* gene. The variant loci are marked by using a green column.

After performing experiments based on the sequenom technology, we randomly selected a few samples from the selected samples to confirm the genotypes for each positive SNP using sanger sequencing performed using ABI3500. The finding exactly coincided with the results obtained from analysis using the sequenom massARRAY technology (Figure 2). The rs2619566 polymorphism in the *CNTN4* gene was a T > C variation (Figure 3A). The rs10260404 polymorphism in the *DPP6* gene was a T > C variation (Figure 3B). The rs79609816 polymorphism in the *INPP5B* gene was an A > T variation (Figure 3C).

### Functional Prediction of Binding Sites and Transcription Factor Binding Sites, Secondary Structure, miRNA and lncRNA Binding for the SNPs Associated With sALS, and Identification of the Genetic Association Between 27 SNPs and sALS Using the Sequenom MassARRAY Technology

**FIGURE 4:**
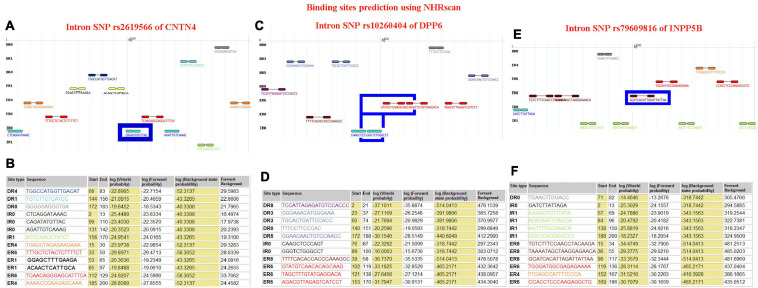
Binding site prediction using NHRscan. **(A)** The IR0 CAGATATGTTAC sequence at positions 99–110 of the *CNTN4* gene is predicted as a binding element, and the SNP is located at the third nucleotide position of the binding fragment. The sites marked using a blue frame indicate the predicted binding fragments. **(B)** Statistical information about the predicted binding sites of the *CNTN4* gene. **(C)** The IR0 GGGTCTGGGCCT sequence at positions 89–100 and the ER6 GTATGTCAACACAGCAAG sequence at positions 102–119 of the *DPP6* gene are predicted as binding fragments, and the SNP is located in the middle of the two binding fragments in the *DPP6* gene. The sites marked using a blue frame indicate the predicted binding fragments. **(D)** Statistical information about the predicted binding sites in the *DPP6* gene. **(E)** The ER8 GCATCACATTAGATTATTAA sequence at positions 98–117 of the *INPP5B* gene is predicted as a binding fragment, and the SNP is located at the fourth nucleotide position of the binding fragment. The sites marked using a blue frame indicate the predicted binding fragments. **(F)** Statistical information about the predicted binding sites in the *INPP5B* gene.

The IR0 element with the sequence CAGATATGTTAC at positions 99–110 of the *CNTN4* gene (Figures 4A,B), the IR0 element with the sequence GGGTCTGGGCCT at positions 89–100, the ER6 element with the sequence GTATGTCAACACAGCAAG at positions 102–119 of the *DPP6* gene (Figures 4C,D), and the ER8 element with the sequence GCATCACATTAGATTATTAA at positions 98–117 of the *INPP5B* gene (Figures 4E,F) were predicted to be binding fragments. The SNP in the *CNTN4* gene associated with sALS was located at the third nucleotide position in the binding fragment of the *CNTN4* gene (Figures 4A,B). The SNP for the *DPP6* gene was located in the middle of the two binding fragments identified in the *DPP6* gene (Figures 4C,D), while the SNP for *INPP5B* was located at the fourth nucleotide position of the binding fragment of the *INPP5B* gene (Figures 4E,F).

**FIGURE 5:**
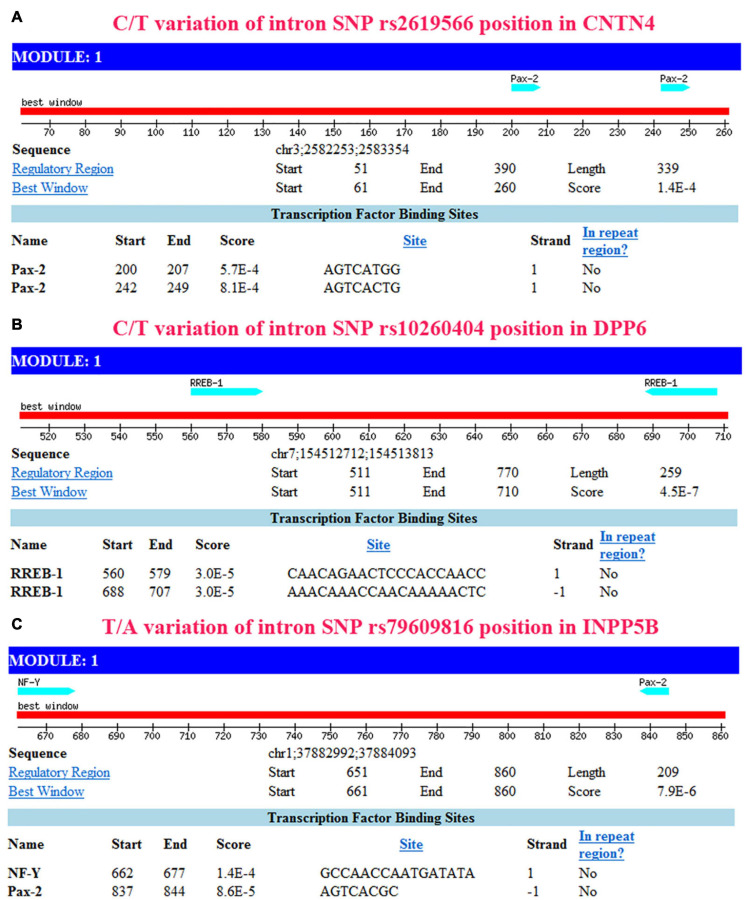
Transcription factor prediction using Mscan. The prediction based on NHRscan and Mscan provided consistent results. **(A)** Transcription factor binding site located between 793 and 800 bp, and between 751 and 758 bp away from the upstream of binding site in *CNTN4*. **(B)** Transcription factor binding site located between 440 and 421 bp, and between 312 and 293 bp away from the upstream of binding site in *DPP6*. **(C)** Transcription factor binding site located between 338 and 323 bp, and between 163 and 156 bp away from the upstream of the binding site in the *INPP5B* gene.

The sites between 793 and 800 bp, and between 751 and 758 bp in the *CNTN4* gene (Figure 5A), the sites between 440 and 421 bp, and between 312 and 293 bp in the *DPP6* gene (Figure 5B), and the sites between 338 and 323 bp, and between 163 and 156 bp in the *INPP5B* gene (Figure 5C) away from the upstream of binding site were hypothesized to be binding sites for transcription factors.

**FIGURE 6:**
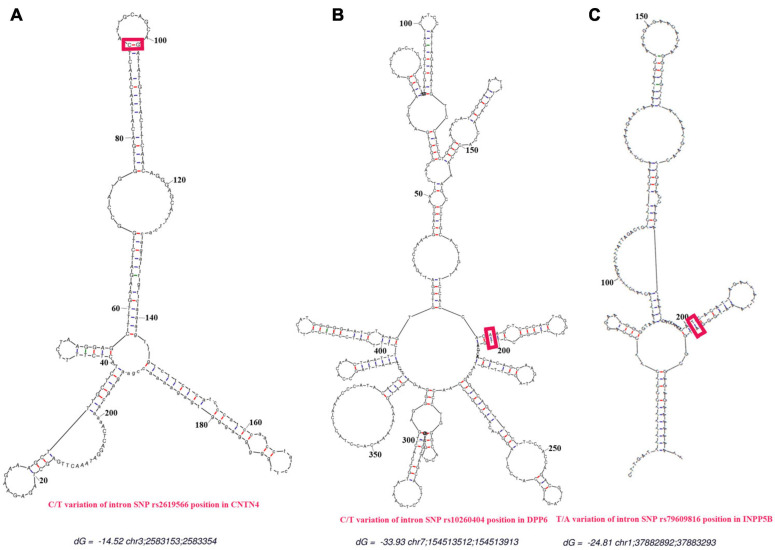
Prediction of secondary structures. The red line represents the predicted binding sites for proteins. The red frames indicate the positions of the SNPs. **(A)** Secondary structure for the rs2619566 T > C variation in the *CNTN4* gene. **(B)** Secondary structure for the rs10260404 T > C variation in the *DPP6* gene. **(C)** Secondary structure for the rs79609816 A > T variation in the *INPP5B* gene. All secondary structures of the binding regions in the *CNTN4*, *DPP6*, and *INPP5B* genes were predicted as hairpin-like structures.

As shown in Figures 6A–C, all secondary structures of the binding site regions in the *CNTN4*, *DPP6*, and *INPP5B* genes formed a hairpin-like structure. No binding sites for miRNA were found after conduction of the miRNA analysis. During the analysis for lncRNA-binding site, the following three sites were predicted to be the possible candidates for lncRNA binding: theENST00000562617 site between 25 and 38 bp upstream of the SNP site, the ENST00000432505 site between 16 and 28 bp away from the downstream, and the ENST00000436078 site between 20 and 32 bp from the SNP in the *CNTN4* gene (Table 5). The ENST00000450077 element between 9 and 21 bp downstream of the SNP site, ENST00000453348 between 28 and 16 bp away from the upstream, ENST00000452622 between 21 and 10 bp at the upstream, ENST00000577700 between 7 and 5 bp at the upstream, and ENST00000418297 between 5 and 21 bp at the downstream in *DPP6* were predicted to be the possible binding candidates of lncRNAs (Table 6). The ENST00000451362 element between 11 and 23 bp downstream of the SNP site, ENST00000591702,ENST00000401018 and ENST00000433505 between 45 and 32 bp away from the upstream of the variation site in *INPP5B* were predicted to be the possible binding candidates for lncRNAs (Table 7).

**TABLE 5:** List of *CNTN4* possible binding candidates of lncRNAs.

ID	Strand	Identity	Alignment
ENST000 00562617 ncrna: lncRNA	Plus/Plus	14/14 (100%)	Query: 12 agatctggccatgg 25
			| | | | | | | | | | | | | | | |
			Sbjct: 525 agatctggccatgg 538
ENST0000 0432505 ncrna: lncRNA	Plus/Plus	13/13 (100%)	Query: 66 agggagcatttca 78
			| | | | | | | | | | | | | | |
			Sbjct: 1769 agggagcatttca 1781
ENST000 00436078 ncrna: lncRNA	Plus/Plus	13/13 (100%)	Query: 70 agcatttcacaga 82
			| | | | | | | | | | | | | | |
			Sbjct: 814 agcatttcacaga 826

**TABLE 6:** List of *DDP6* possible binding candidates of lncRNAs.

ID	Strand	Identity	Alignment
ENST0000 0450077 ncrna: lincRNA	Plus/Minus	13/13 (100%)	Query: 59 aacacagcaagat 71
			| | | | | | | | | | | | | | |
			Sbjct: 353 Aacacagcaagat 341
ENST0000 0453348 ncrna: lincRNA	Plus/Minus	13/13 (100%)	Query: 22 accacaagctccc 34
			| | | | | | | | | | | | | | |
			Sbjct: 627 accacaagctccc 615
ENST000 00452622 ncrna: lincRNA	Plus/Plus	12/12 (100%)	Query: 29 gctcccagtggg 40
			| | | | | | | | | | | | | |
			Sbjct: 824 gctcccagtggg 835
ENST0000 0577700 ncrna: lincRNA	Plus/Plus	13/13 (100%)	Query: 43 ctgggccttgtat 55
			| | | | | | | | | | | | | | |
			Sbjct: 1287 ctgggccttgtat 1299
ENST0000 0418297 ncrna: lincRNA	Plus/Minus	13/13 (100%)	Query: 59 Aacacagcaagat 71
			| | | | | | | | | | | | | | |
			Sbjct: 353 aacacagcaagat 341

**TABLE 7:** List of *INPP5B* possible binding candidates of lncRNAs.

ID	Strand	Identity	Alignment
ENST0000 0451362 ncrna: lincRNA	Plus/Minus	14/14 (100%)	Query: 61 ttattaaatggga 73
			| | | | | | | | | | | | | | | |
			Sbjct: 4602 ttattaaatggga 4590
ENST000 00591702 ncrna: lincRNA	Plus/Minus	14/14 (100%)	Query: 5 acaaggctgtttat 18
			| | | | | | | | | | | | | | | | |
			Sbjct: 1868 acaaggctgtttat 1855
ENST0000 0401018 ncrna: lincRNA	Plus/Minus	14/14 (100%)	Query: 5 acaaggctgtttat 18
			| | | | | | | | | | | | | | | |
			Sbjct: 1810 acaaggctgtttat 1797
ENST0000 0433505 ncrna: lincRNA	Plus/Minus	14/14 (100%)	Query: 5 acaaggctgtttat 18
			| | | | | | | | | | | | | | | |
			Sbjct: 754 acaaggctgtttat 741

## Discussion

Presently, the pathogenesis of sALS is not well understood, while the results of recent investigations suggest that genetic factors may play an important role. To better understand the extent by which genetic factors contribute to the risk of sALS development, it is important to find possible genes or loci that contribute to sALS susceptibility. We conducted a two-stage study in the HACM population consisting of a total of 489 sALS cases and 511 controls, excluding the rapid and slow progressing sALS cases, the early- and late-onset cases, and the sALS cases of atypical clinical manifestations. In the first stage, we performed a pooling GWAS involving 250 sALS cases and 250 controls from the HACM population to screen for possible loci associated with sALS and identified 7 SNPs that showed the most remarkable association with sALS in the HACM population (Xie et al., 2014). Furthermore, we explored previously reported candidate loci associated with the pathogenesis of sALS, and revealed that 20 SNPs were potentially associated with sALS (Table 1; Van Es et al., 2007, 2008; Laaksovirta et al., 2010; Fogh et al., 2014; Van Doormaal et al., 2014). In the second stage, we performed a sequenom massARRAY and DNA sequencing analysis using samples of an independent cohort of 239 sALS cases and 261 controls of HACM ethnicity in order to further identify the relationship with sALS risk for the above-mentioned 27 candidate loci in this study. The results revealed that the rs2619566 in *CNTN4*, the rs10260404 in *DPP6*, and the rs79609816 in *INPP5B* were markedly associated with sALS in the HACM population.

Furthermore, we analyzed the allele and genotype frequencies in sALS cases and controls for the 3 SNPs associated with sALS in the HACM population. All 3 SNPs were intronic polymorphisms. The MAFs of the rs2619566 in the *CNTN4* gene and the rs79609816 in the *INPP5B* gene were significantly higher in the sALS patients than those in the controls. The minor allele frequency of the rs2619566 and the rs79609816 significantly increased the risk of sALS development in the HACM population; the subjects harboring the minor allele C (CC + CT) of rs2619566 and the minor allele T (TT + TA) of rs79609816 exhibited an increased risk of sALS development in comparison with subjects of other genotypes, which indicated that these genotypes were susceptibility factors. The minor allele of rs10260404 in the *DPP6* gene significantly decreased the risk of sALS development in HACM, and the carriers with the minor C allele (CC + CT) of rs10260404 showed a decreased risk of sALS development, thus indicating that the minor allele might be a protective factor (Table 3).

Additionally, we conducted functional predictions for the 3 SNPs in the *CNTN4*, *DPP6*, and *INPP5B* genes. The results of this functional prediction analysis showed that the *CNTN4*, *DPP6*, and *INPP5B* polymorphic regions (3 SNPs) might be binding sites for transcription factors. Possible binding sites for transcription factors were identified at regions more than 700 bp upstream of the *CNTN4* SNPs, more than 400 bp upstream of the *DPP6* SNPs, and more than 300 bp upstream of the *INPP5B* SNPs. The region in the vicinity of more than 20 bp of the *CNTN4* and *DPP6* SNPs, and the vicinity of more than 30 bp of the *INPP5B* SNPs were predicted to be lncRNA non-coding regions, which might play an important role in the regulation of binding proteins (Figures 4–6 and Tables 5–7). These alterations in the *CNTN4*, *DPP6*, and *INPP5B* genes might change or affect their splicing, transcription, and translation, might lead to the generation of abnormal functional and/or structural proteins, and might affect the development of sALS.

*CNTN4* is also known as the *AXCAM* or *BIG-2* gene, and the gene encodes a member of the contactin family of immunoglobulins. CNTNs are axon-associated cell adhesion molecules that demonstrate certain important functions in neuronal network formation and plasticity. The encoded protein is a glycosylphosphatidylinositol-anchored neuronal membrane protein that may play a role in the formation of axon connections in the developing nervous system. The alternative splice results in the generation of multiple transcript variants (Cottrell et al., 2011; Cuoco et al., 2011; Mikulska et al., 2011; Guo et al., 2012; Kaurani et al., 2014). Additionally, the encoded protein of the gene also participates in the function of axon guidance, axonal fasciculation, axonogenesis, brain development, negative regulation of neuronal differentiation, nervous system development, neuronal cell-cell adhesion, neuronal projection development, and regulation of synaptic plasticity (Fernandez et al., 2004; Oguro-Ando et al., 2017).

**FIGURE 7:**
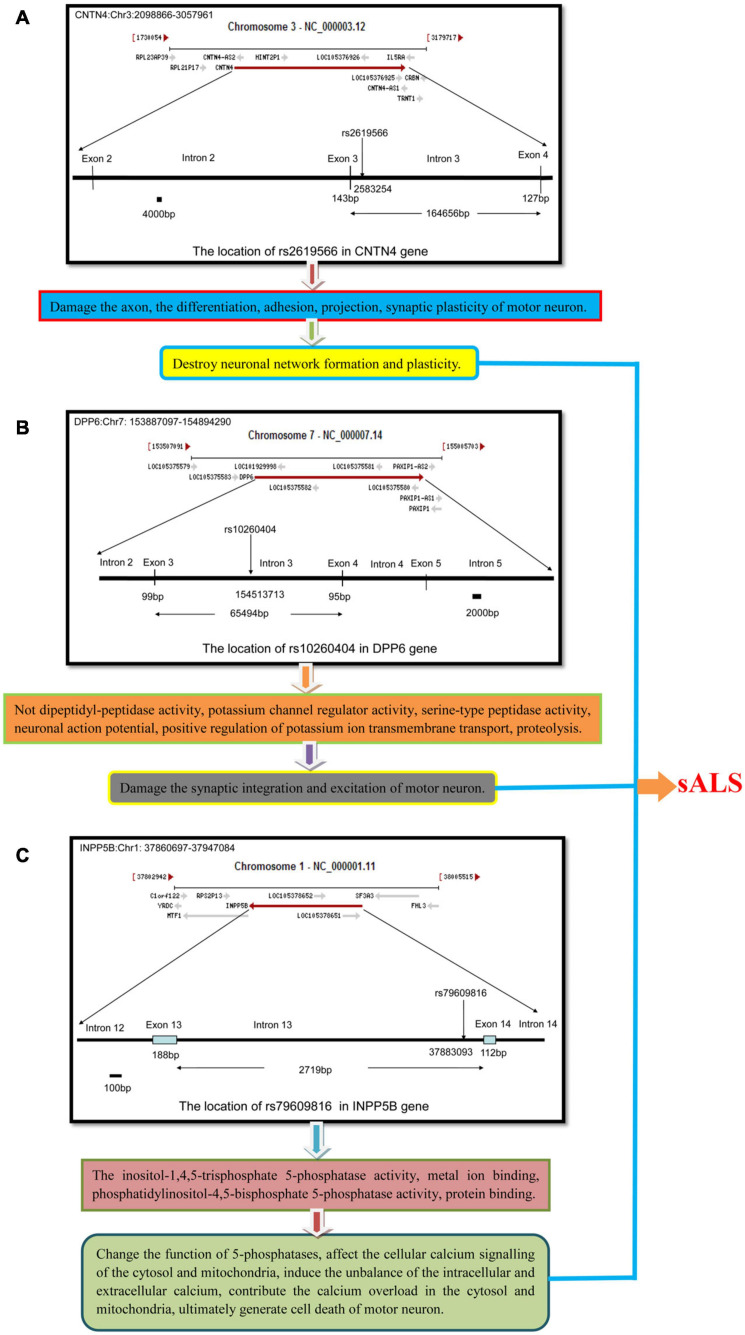
The schematic diagram about the mechanisms that the polymorphisms of rs2619566 in *CNTN4*, rs10260404 in *DPP6* and rs79609816 in *INPP5B* might result in sALS from HACM. The pathogenesis of sALS might be a mechanism of multiple genes and loci participation through a series of complex pathophysiologic pathways. **(A)** The polymorphisms of rs2619566 in *CNTN4* might be associated with destroy of neuronal network formation and plasticity. **(B)** The polymorphisms of rs10260404 in *DPP6* might be related to damaging synaptic integration and excitation of neuron. **(C)** The polymorphisms of rs79609816 in *INPP5B* might induce the calcium disorder in cytosol and mitochondria, contributed to neuron death through a series of pathophysiological processes.

*CNTN4* alteration may contribute toward the infliction of damage of the neuronal axons, as well as affect neuron differentiation, adhesion, projections, and synaptic plasticity. In our study, we found that the subjects harboring the minor C allele (CC + CT) of the rs2619566 polymorphism in the *CNTN4* gene had a significantly increased risk of developing sALS. The pathogenesis mechanisms related to sALS development may rely on the *CNTN4* gene harboring the minor C allele (CC + CT) that results in rs2619566 changes or affects splicing, transcription, or translation of the *CNTN4* gene, thereby generating an abnormal *CNTN4* protein, which induces abnormalities in differentiation, adhesion, projection, synaptic plasticity, axonal guidance, and fasciculation of motor neurons, contributing to the development of lesions and disturbance in neuronal network formation and plasticity, subsequently resulting in the development of sALS (Figure 7A).

*DPP6* also is known as *VF2 DPPX* and *MRD33*. This gene encodes a single-pass type II membrane protein that is a member of the peptidase S9B family of serine proteases. This protein has no detectable protease activity, and this is most likely attributed to the absence of the conserved serine residue normally present in the catalytic domain of serine proteases. However, it does bind specific voltage-gated potassium channels and alters their expression and biophysical properties. Variations in this gene may be associated with susceptibility to sALS (Cronin et al., 2008, 2009; Del Bo et al., 2008; Kwee et al., 2012). Only 9 published studies have reported on the association between *DPP6* and sALS thus far, and of these 9 studies, only 2 independent studies were conducted in the Chinese population (Li et al., 2009; Chen et al., 2012), and both studies suggested the absence of association of the rs10260404 variant in *DPP6* with sALS development in the Chinese population. However, the other 7 studies performed in European populations including Italian, Dutch, Polish, and Irish, and Americans, reported results that were dramatically different; 5 of the 7 studies suggested that *DPP6* was a candidate gene for sALS, and reported several candidate loci associated with sALS (Cronin et al., 2008, 2009; Del Bo et al., 2008; Kwee et al., 2012), while 2 of the 7 studies indicated that mutations in these genes were unlikely to be associated with sALS (Chiò et al., 2009; Daoud et al., 2010). Two independent studies suggested the absence of association of the rs10260404 variant in DPP6 with sALS development in the Chinese population (Li et al., 2009; Chen et al., 2012). Actually, in our previous GWAS study, there was no correlation between the rs10260404 variant in DPP6 and sALS yet (Xie et al., 2014). But in this validation study, it showed that the rs10260404 variant in DPP6 was associated with sALS in Chinese Han populations. The contrary conclusions might be due to the differences of their ethnicity or population background or sample size. The further study need conduct in the larger sample size and different ethnicity and population background.

An alternative splicing of *DPP6* results in the generation of multiple transcript variants (Lin et al., 2014). *DPP6* is critical for the synaptic integration and excitation of neurons (Wolf et al., 2014). The main functions of *DPP6* include the not dipeptidyl-peptidase activity, potassium channel regulator activity, serine-type peptidase activity (Yokotani et al., 1993), neuronal action potential regulation, positive regulation of potassium ion transmembrane transport, and proteolysis (Soh and Goldstein, 2008). Our results revealed that carriers of the minor allele C (CC + CT) of the rs10260404 polymorphism in the *DPP6* gene had an decreased risk of developing sALS, suggesting that presence of the minor C allele might change or affect the splicing, transcription, or translation of the *DPP6* gene, which might be responsible for an decreased risk of sALS development. According to the existing knowledge on the *DPP6* function, the protective pathogenesis associated with sALS might be attributable to an abnormal alteration of the protein activity due to the rs10260404 polymorphism-associated changes improving the synaptic integration and excitation of motor neurons. Meanwhile, the known functions of *DPP6* might be involved in the process, subsequently contributing to the decreased risk of sALS (Figure 7B).

*INPP5B* is also known as 5PTase. This gene encodes a member of a family of inositol polyphosphate-5-phosphatases (5-phosphatases) (Ross et al., 1991). The enzyme functions in the regulation of calcium signaling by inactivating inositol phosphates (IPs). The encoded protein is localized in the cytosol and mitochondria, and establishes association with membranes through an isoprenyl modification near the C-terminus. Cellular calcium signaling is controlled by the production of IPs and by phospholipase C in response to extracellular signals. The IPs signaling molecules are inactivated by activities of a family of 5-phosphatases. Several alternatively spliced transcript variants of this gene have been described, but the full-length transcript features of a few of these variants have not been determined. The main functions of *INPP5B* include inositol-1,4,5-trisphosphate 5-phosphatase activity, metal ion binding, phosphatidylinositol-4,5-bisphosphate 5-phosphatase activity, and protein binding (Jefferson and Majerus, 1995; Noakes et al., 2011). In the results of the present study, the MAFs for the rs79609816 polymorphism in the *INPP5B* gene were higher in the sALS patients than those in the controls, and subjects harboring the minor allele T (TT + TA) had a significantly higher risk of developing sALS. We hypothesize that the minor allele T of the *INPP5B* rs79609816 polymorphism may affect the splicing, transcription, or translation of the *INPP5B* gene, that may further generate an abnormal *INPP5B* protein, which results in an increased risk for sALS development and represents a susceptibility factor for this disease. The alteration due to the *INPP5B* rs79609816 variant might change the function of 5-phosphatases, affect cellular calcium signaling in the cytosol and mitochondria, induce an imbalance in the intracellular and extracellular calcium levels, contribute to calcium overload in the cytosol and mitochondria, and ultimately result in the death of motor neurons (Figure 7C). In conclusion, our study found that 3 polymorphisms, namely the *CNTN4* rs2619566, the *DPP6* rs10260404, and the rs79609816, were significantly associated with sALS development in the HACM population (Figure 7).

Based on the currently known functions of the *CNTN4*, *DPP6*, and *INPP5B* genes, it is possible to postulate that *CNTN4* rs2619566 variants may inflict damage on the neuronal network formation and plasticity of motor neurons, that *DPP6* rs10260404 variants may improve the synaptic integration and excitation of motor neurons, and that *INPP5B* rs79609816 variants may destroy cellular calcium signaling of the cytosol and mitochondria in motor neurons, ultimately influence the sALS development. In the process, the function of axon guidance, axonal fasciculation, axonogenesis, negative regulation of neuron differentiation, nervous system development, neuron cell-cell adhesion, neuron projection development, regulation of synaptic plasticity, not dipeptidyl-peptidase activity, potassium channel regulator activity, serine-type peptidase activity, neuronal action potential, positive regulation of potassium ion transmembrane transport, proteolysis, inositol-1,4,5-trisphosphate-5-phosphatase activity, metal ion binding, phosphatidylinositol-4,5-bisphosphate 5-phosphatase activity, and protein binding may all directly or indirectly participate in the development of sALS (Figure 7).

Our data are consistent with those obtained using a model of sALS pathogenesis that involves a series of complex pathophysiologic processes attributed to the presence of multiple genes and loci. These polymorphisms might change or affect splicing, transcription, and translation, resulting in the production of abnormal *CNTN4*, *DPP6*, and *INPP5B* proteins, which might inflict damage or disrupt or improve neuronal network formation and plasticity, the synaptic integration and excitation, and the cellular calcium signaling of the cytosol and mitochondria of motor neurons, ultimately influencing the development of sALS. Our study further suggests that multiple genes and loci participate in the susceptibility to sALS. Our results provide valuable data for conducting further studies on the elucidation of the pathogenesis of sALS.

## Data Availability Statement

The original contributions presented in the study are included in the article/supplementary material, further inquiries can be directed to the corresponding author/s.

## Ethics Statement

The study was approved by the Institutional Review Board of the Hospital Human Ethics Committee of The First Affiliated Hospital of Nanchang University and was conducted in accordance with the approved guidelines and regulations. The patients/participants provided their written informed consent to participate in this study.

## Author Contributions

JZ and RX conceived, designed, and performed the experiments, analyzed the data, and wrote the manuscript. XZ, YD, and HN conceived and designed the experiments. JZ, WQ, and FH performed the experiments, analyzed the data, and were the jointed first authors. XZ, YD, HN, and RX contributed reagents, materials, tools, and services and were the co-corresponding authors. RX was the corresponding author. All authors were involved in the drafting, critical revision, read, and final approval of the manuscript for publication and agreed to be accountable for all aspects of the work in ensuring that questions related to the accuracy or integrity of any part of the work are appropriately investigated and resolved and contributed significantly to this research and in the preparation of the manuscript.

## Conflict of Interest

The authors declare that the research was conducted in the absence of any commercial or financial relationships that could be construed as a potential conflict of interest.

## Publisher’s Note

All claims expressed in this article are solely those of the authors and do not necessarily represent those of their affiliated organizations, or those of the publisher, the editors and the reviewers. Any product that may be evaluated in this article, or claim that may be made by its manufacturer, is not guaranteed or endorsed by the publisher.

## Footnotes

1
www.ensembl.org

## Abbreviations

sALS: Sporadic Amyotrophic Lateral Sclerosis
HACM: Han Ancestry of Chinese Mainland
CNS: Central Nervous System
fALS: Familial ALS
*CNTN4*: Contactin 4
*DPP6*: Dipeptidyl-Peptidase 6
*INPP5B*: Inositol Polyphosphate-5-Phosphatases B/2
SNPs: Single Nucleotide Polymorphisms
GWAS: Genome-Wide Association Study
PCR: Polymerase Chain Reaction
SAP: Shrimp Alkaline Phosphatase
MAF: Minor Allele Frequency
OR: Odds Ratios
CI: Confidence Intervals.
